# {*N*,*N*′-Bis[1-(2-pyrid­yl)ethyl­idene]ethane-1,2-diamine-κ^4^ *N*,*N*′,*N*′′,*N*′′′}(thio­cyanato-κ*N*)zinc(II) perchlorate

**DOI:** 10.1107/S1600536810021628

**Published:** 2010-06-16

**Authors:** Fu-Ming Wang

**Affiliations:** a Department of Chemistry, Dezhou University, Dezhou Shandong 253023, People’s Republic of China

## Abstract

In the title compound, [Zn(NCS)(C_16_H_18_N_4_)]ClO_4_, the Zn^II^ atom is five-coordinated by four N atoms of the Schiff base ligand *N*,*N*′-bis­[1-(2-pyrid­yl)ethyl­idene]ethane-1,2-diamine in the basal plane, and by the N atom of a thio­cyanate ligand at the apical position, forming a distorted square-pyramidal geometry. The r.m.s. deviation from a plane through the four N atoms of the Schiff base is 0.015 (3) Å, and the deviation of the Ni atom from that plane is 0.591 (2) Å. Bond lengths are comparable with those observed in similar zinc(II) complexes with Schiff bases. The two methyl­ene C atoms of the ethane-1,2-diamine bridge of the Schiff base ligand are disordered over two sites with occupancies of 0.587 (3) and 0.413 (3).

## Related literature

For background to Schiff base compounds and their applications, see: Ruck & Jacobsen (2002 ▶); Mukhopadhyay *et al.* (2003 ▶); Polt *et al.* (2003 ▶); Mukherjee *et al.* (2001 ▶). For complexes derived from *N*,*N*′-bis­(1-(pyridin-2-yl)ethyl­idene)ethane-1,2-diamine, see: Gourbatsis *et al.* (1998 ▶); Louloudi *et al.* (1999 ▶); Karmakar *et al.* (2002 ▶); Banerjee *et al.* (2004 ▶). For bond lengths in similar zinc(II) complexes with Schiff bases, see: Ghosh *et al.* (2006 ▶); Chen *et al.* (2005 ▶). For the synthesis of the Schiff base ligand, see: Gourbatsis *et al.* (1990 ▶).
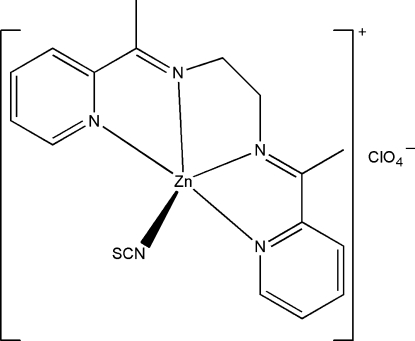


## Experimental

### 

#### Crystal data

[Zn(NCS)(C_16_H_18_N_4_)]ClO_4_
*M* *_r_* = 489.24
Monoclinic, 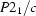

*a* = 8.685 (2) Å
*b* = 13.963 (3) Å
*c* = 17.374 (2) Å
β = 99.690 (3)°
*V* = 2076.9 (7) Å^3^
*Z* = 4
Mo *K*α radiation
μ = 1.45 mm^−1^
*T* = 298 K
0.32 × 0.30 × 0.30 mm

#### Data collection

Bruker SMART CCD area-detector diffractometer
Absorption correction: multi-scan (*SADABS*; Sheldrick, 1996 ▶) *T*_min_ = 0.655, *T*_max_ = 0.671
16681 measured reflections
4529 independent reflections
2534 reflections with *I* > 2σ(*I*)
*R*_int_ = 0.056

#### Refinement

*R*[*F* ^2^ > 2σ(*F* ^2^)] = 0.056
*wR*(*F* ^2^) = 0.170
*S* = 1.02
4529 reflections
282 parameters
48 restraints
H-atom parameters constrained
Δρ_max_ = 0.50 e Å^−3^
Δρ_min_ = −0.66 e Å^−3^

### 

Data collection: *SMART* (Bruker, 1998 ▶); cell refinement: *SAINT* (Bruker, 1998 ▶); data reduction: *SAINT*; program(s) used to solve structure: *SHELXS97* (Sheldrick, 2008 ▶); program(s) used to refine structure: *SHELXL97* (Sheldrick, 2008 ▶); molecular graphics: *SHELXTL* (Sheldrick, 2008 ▶); software used to prepare material for publication: *SHELXTL*.

## Supplementary Material

Crystal structure: contains datablocks global, I. DOI: 10.1107/S1600536810021628/sj5017sup1.cif

Structure factors: contains datablocks I. DOI: 10.1107/S1600536810021628/sj5017Isup2.hkl

Additional supplementary materials:  crystallographic information; 3D view; checkCIF report

## Figures and Tables

**Table:** 

Zn1—N5	1.982 (5)
Zn1—N3	2.099 (4)
Zn1—N1	2.100 (4)
Zn1—N2	2.102 (4)
Zn1—N4	2.115 (4)

**Table:** 

N5—Zn1—N3	109.08 (17)
N5—Zn1—N1	105.95 (16)
N3—Zn1—N1	141.17 (14)
N5—Zn1—N2	110.56 (19)
N3—Zn1—N2	75.37 (15)
N1—Zn1—N2	77.12 (15)
N5—Zn1—N4	101.91 (17)
N3—Zn1—N4	77.11 (14)
N1—Zn1—N4	111.48 (15)
N2—Zn1—N4	142.71 (17)

**Fig. 1.**
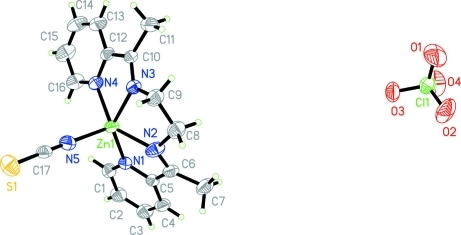
The asymmetric unit of the title compound, showing the atom-numbering scheme. Displacement ellipsoids are drawn at the 30% probability level. Only the major component of the disordered group is shown.

## supplementary crystallographic information

### Comment

Metal complexes with Schiff bases have been known since 1840. The Schiff bases and their complexes have played an important role in the development of coordination chemistry, biological and material sciences (Ruck & Jacobsen, 2002; Mukhopadhyay *et al.*, 2003; Polt *et al.*, 2003; Mukherjee *et al.*, 2001). Several complexes derived from *N*,*N*'-bis(1-(pyridin-2-yl)ethylidene)ethane-1,2-diamine have been reported (Gourbatsis *et al.*, 1998; Louloudi *et al.*, 1999; Karmakar *et al.*, 2002; Banerjee *et al.*, 2004). In this paper, the title new zinc(II) complex is reported.

The title compound consists of a mononuclear zinc(II) complex cation and a perchlorate anion, Fig. 1. The Zn^II^ atom is five-coordinated by four N atoms of the Schiff base ligand *N*,*N*'-bis(1-(pyridin-2-yl)ethylidene)ethane-1,2-diamine, and by one N atom of a thiocyanate ligand, forming a square pyramidal geometry. The coordinate bond lengths (Table 1) are comparable to those observed in other similar zinc(II) complexes with Schiff bases (Ghosh *et al.*, 2006; Chen *et al.*, 2005).

### Experimental

The Schiff base ligand *N*,*N*'-bis(1-(pyridin-2-yl)ethylidene)ethane-1,2-diamine was synthesized according to the literature method (Gourbatsis *et al.*, 1990). To a stirred methanol solution of the Schiff base ligand (1.0 mmol, 0.266 g) was added a methanol solution of zinc(II) perchlorate (1.0 mmol, 0.390 g) and ammonium thiocyanate (1.0 mmol, 0.076 g). The mixture was boiled under reflux for 2 h, then cooled to room temperature. Colourless block-like single crystals, suitable for X-ray diffraction, were formed after slow evaporation of the solution in air for a few days.

### Refinement

Hydrogen atoms were placed in geometrically idealized positions and constrained to ride on their parent atoms, with C–H distances of 0.93–0.97 Å, and with *U*_iso_(H) set at 1.2*U*_eq_(C) and 1.5*U*_eq_(C_methyl_). The C8 and C9 atoms are disordered over two sites with occupancies of 0.587 (3) and 0.413 (3).

### Crystal data

**Table:**

[Zn(NCS)(C_16_H_18_N_4_)]ClO_4_	*F*(000) = 1000
*M**_r_* = 489.24	*D*_x_ = 1.565 Mg m^−^^3^
Monoclinic, *P*2_1_/*c*	Mo *K*α radiation, λ = 0.71073 Å
Hall symbol: -P 2ybc	Cell parameters from 2719 reflections
*a* = 8.685 (2) Å	θ = 2.4–25.0°
*b* = 13.963 (3) Å	µ = 1.45 mm^−^^1^
*c* = 17.374 (2) Å	*T* = 298 K
β = 99.690 (3)°	Block, colourless
*V* = 2076.9 (7) Å^3^	0.32 × 0.30 × 0.30 mm
*Z* = 4	

### Data collection

**Table:**

Bruker SMART CCD area-detector diffractometer	4529 independent reflections
Radiation source: fine-focus sealed tube	2534 reflections with *I* > 2σ(*I*)
graphite	*R*_int_ = 0.056
ω scan	θ_max_ = 27.0°, θ_min_ = 2.4°
Absorption correction: multi-scan (*SADABS*; Sheldrick, 1996)	*h* = −11→10
*T*_min_ = 0.655, *T*_max_ = 0.671	*k* = −17→16
16681 measured reflections	*l* = −22→22

### Refinement

**Table:**

Refinement on *F*^2^	Primary atom site location: structure-invariant direct methods
Least-squares matrix: full	Secondary atom site location: difference Fourier map
*R*[*F*^2^ > 2σ(*F*^2^)] = 0.056	Hydrogen site location: inferred from neighbouring sites
*wR*(*F*^2^) = 0.170	H-atom parameters constrained
*S* = 1.02	*w* = 1/[σ^2^(*F*_o_^2^) + (0.0792*P*)^2^ + 0.7526*P*] where *P* = (*F*_o_^2^ + 2*F*_c_^2^)/3
4529 reflections	(Δ/σ)_max_ < 0.001
282 parameters	Δρ_max_ = 0.50 e Å^−^^3^
48 restraints	Δρ_min_ = −0.66 e Å^−^^3^

### Special details

**Table:**

Geometry. All e.s.d.'s (except the e.s.d. in the dihedral angle between two l.s. planes) are estimated using the full covariance matrix. The cell e.s.d.'s are taken into account individually in the estimation of e.s.d.'s in distances, angles and torsion angles; correlations between e.s.d.'s in cell parameters are only used when they are defined by crystal symmetry. An approximate (isotropic) treatment of cell e.s.d.'s is used for estimating e.s.d.'s involving l.s. planes.
Refinement. Refinement of *F*^2^ against ALL reflections. The weighted *R*-factor *wR* and goodness of fit *S* are based on *F*^2^, conventional *R*-factors *R* are based on *F*, with *F* set to zero for negative *F*^2^. The threshold expression of *F*^2^ > σ(*F*^2^) is used only for calculating *R*-factors(gt) *etc*. and is not relevant to the choice of reflections for refinement. *R*-factors based on *F*^2^ are statistically about twice as large as those based on *F*, and *R*- factors based on ALL data will be even larger.

### Fractional atomic coordinates and isotropic or equivalent isotropic displacement parameters (Å^2^)

**Table:**

	*x*	*y*	*z*	*U*_iso_*/*U*_eq_	Occ. (<1)
Zn1	0.40268 (7)	0.18336 (3)	0.23821 (3)	0.0532 (2)	
Cl1	0.13446 (17)	0.32198 (10)	0.93392 (9)	0.0761 (4)	
S1	0.80304 (19)	0.18105 (12)	0.08648 (10)	0.0888 (5)	
O1	0.1863 (9)	0.2376 (4)	0.9693 (3)	0.170 (3)	
O2	0.2281 (6)	0.3965 (4)	0.9651 (3)	0.145 (2)	
O3	0.1346 (6)	0.3155 (3)	0.8524 (2)	0.0975 (14)	
O4	−0.0172 (5)	0.3397 (4)	0.9480 (3)	0.1246 (19)	
N1	0.2700 (4)	0.3093 (2)	0.2175 (2)	0.0516 (9)	
N4	0.2661 (4)	0.0604 (3)	0.2040 (2)	0.0547 (10)	
N5	0.5483 (6)	0.1840 (3)	0.1618 (3)	0.0725 (13)	
C1	0.1666 (6)	0.3311 (4)	0.1535 (3)	0.0640 (13)	
H1	0.1435	0.2853	0.1144	0.077*	
C2	0.0928 (6)	0.4194 (4)	0.1434 (3)	0.0724 (15)	
H2	0.0234	0.4328	0.0978	0.087*	
C3	0.1231 (7)	0.4857 (4)	0.2009 (4)	0.0762 (16)	
H3	0.0728	0.5447	0.1959	0.091*	
C4	0.2296 (6)	0.4645 (3)	0.2672 (3)	0.0673 (14)	
H4	0.2531	0.5097	0.3067	0.081*	
C5	0.3015 (6)	0.3754 (3)	0.2744 (3)	0.0524 (11)	
C6	0.4210 (6)	0.3474 (3)	0.3435 (3)	0.0608 (13)	
C7	0.4484 (8)	0.4109 (4)	0.4138 (3)	0.0881 (19)	
H7A	0.3514	0.4216	0.4320	0.132*	
H7B	0.4902	0.4710	0.4003	0.132*	
H7C	0.5212	0.3808	0.4543	0.132*	
N2	0.4901 (5)	0.2687 (3)	0.3355 (3)	0.0713 (12)	0.59 (3)
N3	0.4830 (4)	0.0850 (3)	0.3273 (2)	0.0537 (10)	0.59 (3)
C8	0.573 (2)	0.2217 (8)	0.4079 (7)	0.076 (4)	0.59 (3)
H8A	0.5077	0.2231	0.4481	0.092*	0.59 (3)
H8B	0.6691	0.2557	0.4272	0.092*	0.59 (3)
C9	0.6085 (18)	0.1192 (11)	0.3889 (10)	0.073 (6)	0.59 (3)
H9A	0.7083	0.1159	0.3709	0.088*	0.59 (3)
H9B	0.6141	0.0795	0.4351	0.088*	0.59 (3)
N2'	0.4901 (5)	0.2687 (3)	0.3355 (3)	0.0713 (12)	0.41 (3)
N3'	0.4830 (4)	0.0850 (3)	0.3273 (2)	0.0537 (10)	0.41 (3)
C8'	0.6313 (16)	0.2226 (10)	0.3782 (13)	0.058 (5)	0.41 (3)
H8'A	0.6739	0.2587	0.4247	0.070*	0.41 (3)
H8'B	0.7109	0.2158	0.3456	0.070*	0.41 (3)
C9'	0.571 (3)	0.1262 (15)	0.3990 (8)	0.058 (6)	0.41 (3)
H9'A	0.6570	0.0846	0.4201	0.070*	0.41 (3)
H9'B	0.5035	0.1335	0.4379	0.070*	0.41 (3)
C10	0.4124 (6)	0.0058 (3)	0.3271 (3)	0.0531 (12)	
C11	0.4348 (8)	−0.0665 (4)	0.3924 (3)	0.0878 (19)	
H11A	0.5251	−0.0497	0.4299	0.132*	
H11B	0.4493	−0.1290	0.3716	0.132*	
H11C	0.3442	−0.0670	0.4174	0.132*	
C12	0.2956 (5)	−0.0134 (3)	0.2555 (3)	0.0518 (11)	
C13	0.2222 (6)	−0.1010 (4)	0.2399 (3)	0.0714 (15)	
H13	0.2424	−0.1509	0.2756	0.086*	
C14	0.1198 (7)	−0.1136 (4)	0.1718 (4)	0.089 (2)	
H14	0.0677	−0.1716	0.1614	0.107*	
C15	0.0947 (7)	−0.0406 (5)	0.1193 (4)	0.093 (2)	
H15	0.0289	−0.0491	0.0717	0.112*	
C16	0.1684 (6)	0.0465 (4)	0.1376 (3)	0.0775 (16)	
H16	0.1487	0.0968	0.1022	0.093*	
C17	0.6548 (7)	0.1821 (3)	0.1302 (3)	0.0544 (12)	

### Atomic displacement parameters (Å^2^)

**Table:**

	*U*^11^	*U*^22^	*U*^33^	*U*^12^	*U*^13^	*U*^23^
Zn1	0.0591 (4)	0.0415 (3)	0.0582 (4)	−0.0002 (3)	0.0079 (3)	0.0004 (2)
Cl1	0.0691 (9)	0.0799 (10)	0.0767 (9)	−0.0045 (8)	0.0053 (7)	−0.0098 (8)
S1	0.0678 (10)	0.1090 (13)	0.0936 (12)	0.0072 (9)	0.0252 (9)	0.0140 (9)
O1	0.247 (6)	0.129 (4)	0.128 (4)	0.090 (4)	0.019 (4)	0.038 (4)
O2	0.128 (4)	0.170 (5)	0.139 (4)	−0.084 (4)	0.031 (3)	−0.071 (4)
O3	0.117 (3)	0.115 (3)	0.063 (2)	−0.004 (3)	0.023 (2)	−0.015 (2)
O4	0.054 (3)	0.207 (6)	0.119 (4)	0.000 (3)	0.031 (3)	−0.013 (3)
N1	0.058 (2)	0.045 (2)	0.052 (2)	0.0013 (18)	0.0111 (18)	0.0031 (17)
N4	0.050 (2)	0.050 (2)	0.062 (2)	0.0005 (18)	0.0044 (19)	−0.0043 (19)
N5	0.088 (3)	0.055 (3)	0.080 (3)	0.003 (2)	0.032 (3)	0.009 (2)
C1	0.072 (3)	0.062 (3)	0.057 (3)	0.003 (3)	0.009 (3)	0.001 (2)
C2	0.077 (4)	0.062 (3)	0.077 (4)	0.018 (3)	0.008 (3)	0.017 (3)
C3	0.085 (4)	0.058 (3)	0.088 (4)	0.022 (3)	0.017 (3)	0.013 (3)
C4	0.076 (4)	0.043 (3)	0.086 (4)	0.001 (3)	0.024 (3)	−0.005 (3)
C5	0.057 (3)	0.045 (3)	0.058 (3)	−0.009 (2)	0.018 (2)	0.001 (2)
C6	0.068 (3)	0.043 (3)	0.069 (3)	−0.012 (2)	0.002 (3)	−0.001 (2)
C7	0.115 (5)	0.061 (3)	0.079 (4)	−0.008 (3)	−0.009 (3)	−0.017 (3)
N2	0.064 (3)	0.053 (3)	0.088 (3)	−0.001 (2)	−0.015 (2)	−0.004 (2)
N3	0.055 (2)	0.049 (2)	0.055 (2)	−0.0005 (19)	0.0049 (18)	−0.0021 (18)
C8	0.073 (8)	0.071 (6)	0.080 (7)	−0.002 (5)	−0.002 (6)	−0.008 (5)
C9	0.080 (9)	0.063 (8)	0.071 (8)	0.004 (6)	−0.003 (6)	−0.007 (5)
N2'	0.064 (3)	0.053 (3)	0.088 (3)	−0.001 (2)	−0.015 (2)	−0.004 (2)
N3'	0.055 (2)	0.049 (2)	0.055 (2)	−0.0005 (19)	0.0049 (18)	−0.0021 (18)
C8'	0.044 (7)	0.058 (7)	0.069 (8)	−0.001 (6)	0.001 (6)	−0.010 (6)
C9'	0.068 (9)	0.051 (9)	0.051 (8)	0.005 (7)	−0.003 (7)	0.004 (6)
C10	0.065 (3)	0.039 (2)	0.057 (3)	0.008 (2)	0.017 (2)	0.000 (2)
C11	0.129 (6)	0.055 (3)	0.076 (4)	−0.006 (3)	0.008 (4)	0.009 (3)
C12	0.048 (3)	0.043 (3)	0.068 (3)	0.004 (2)	0.020 (2)	−0.007 (2)
C13	0.065 (4)	0.050 (3)	0.098 (4)	−0.007 (3)	0.013 (3)	−0.006 (3)
C14	0.076 (4)	0.058 (4)	0.129 (6)	−0.015 (3)	0.001 (4)	−0.021 (4)
C15	0.077 (4)	0.085 (5)	0.104 (5)	−0.011 (4)	−0.023 (4)	−0.023 (4)
C16	0.071 (4)	0.069 (4)	0.084 (4)	0.001 (3)	−0.011 (3)	−0.004 (3)
C17	0.065 (3)	0.038 (2)	0.058 (3)	0.001 (2)	0.002 (3)	0.007 (2)

### Geometric parameters (Å, °)

**Table:**

Zn1—N5	1.982 (5)	C7—H7C	0.9600
Zn1—N3	2.099 (4)	N2—C8	1.492 (8)
Zn1—N1	2.100 (4)	N3—C10	1.264 (5)
Zn1—N2	2.102 (4)	N3—C9	1.472 (8)
Zn1—N4	2.115 (4)	C8—C9	1.513 (10)
Cl1—O1	1.370 (5)	C8—H8A	0.9700
Cl1—O2	1.374 (4)	C8—H8B	0.9700
Cl1—O4	1.401 (4)	C9—H9A	0.9700
Cl1—O3	1.419 (4)	C9—H9B	0.9700
S1—C17	1.601 (6)	C8'—C9'	1.512 (10)
N1—C1	1.340 (6)	C8'—H8'A	0.9700
N1—C5	1.347 (5)	C8'—H8'B	0.9700
N4—C16	1.327 (6)	C9'—H9'A	0.9700
N4—C12	1.359 (6)	C9'—H9'B	0.9700
N5—C17	1.153 (7)	C10—C12	1.492 (6)
C1—C2	1.387 (7)	C10—C11	1.508 (7)
C1—H1	0.9300	C11—H11A	0.9600
C2—C3	1.355 (7)	C11—H11B	0.9600
C2—H2	0.9300	C11—H11C	0.9600
C3—C4	1.382 (7)	C12—C13	1.385 (6)
C3—H3	0.9300	C13—C14	1.368 (8)
C4—C5	1.388 (6)	C13—H13	0.9300
C4—H4	0.9300	C14—C15	1.360 (8)
C5—C6	1.501 (7)	C14—H14	0.9300
C6—N2	1.271 (6)	C15—C16	1.387 (7)
C6—C7	1.495 (7)	C15—H15	0.9300
C7—H7A	0.9600	C16—H16	0.9300
C7—H7B	0.9600		
			
N5—Zn1—N3	109.08 (17)	C6—N2—Zn1	117.7 (3)
N5—Zn1—N1	105.95 (16)	C8—N2—Zn1	119.2 (5)
N3—Zn1—N1	141.17 (14)	C10—N3—C9	125.9 (9)
N5—Zn1—N2	110.56 (19)	C10—N3—Zn1	118.0 (3)
N3—Zn1—N2	75.37 (15)	C9—N3—Zn1	115.9 (8)
N1—Zn1—N2	77.12 (15)	N2—C8—C9	108.5 (11)
N5—Zn1—N4	101.91 (17)	N2—C8—H8A	110.0
N3—Zn1—N4	77.11 (14)	C9—C8—H8A	110.0
N1—Zn1—N4	111.48 (15)	N2—C8—H8B	110.0
N2—Zn1—N4	142.71 (17)	C9—C8—H8B	110.0
O1—Cl1—O2	110.3 (5)	H8A—C8—H8B	108.4
O1—Cl1—O4	108.8 (4)	N3—C9—C8	108.2 (9)
O2—Cl1—O4	108.0 (3)	N3—C9—H9A	110.1
O1—Cl1—O3	109.7 (3)	C8—C9—H9A	110.1
O2—Cl1—O3	110.0 (3)	N3—C9—H9B	110.1
O4—Cl1—O3	110.1 (3)	C8—C9—H9B	110.1
C1—N1—C5	118.6 (4)	H9A—C9—H9B	108.4
C1—N1—Zn1	127.1 (3)	C9'—C8'—H8'A	111.3
C5—N1—Zn1	114.2 (3)	C9'—C8'—H8'B	111.3
C16—N4—C12	118.9 (4)	H8'A—C8'—H8'B	109.2
C16—N4—Zn1	127.5 (4)	C8'—C9'—H9'A	110.2
C12—N4—Zn1	113.3 (3)	C8'—C9'—H9'B	110.2
C17—N5—Zn1	166.6 (5)	H9'A—C9'—H9'B	108.5
N1—C1—C2	122.5 (5)	N3—C10—C12	114.9 (4)
N1—C1—H1	118.7	N3—C10—C11	125.6 (5)
C2—C1—H1	118.7	C12—C10—C11	119.4 (4)
C3—C2—C1	119.1 (5)	C10—C11—H11A	109.5
C3—C2—H2	120.5	C10—C11—H11B	109.5
C1—C2—H2	120.5	H11A—C11—H11B	109.5
C2—C3—C4	119.2 (5)	C10—C11—H11C	109.5
C2—C3—H3	120.4	H11A—C11—H11C	109.5
C4—C3—H3	120.4	H11B—C11—H11C	109.5
C3—C4—C5	119.7 (5)	N4—C12—C13	120.8 (4)
C3—C4—H4	120.1	N4—C12—C10	116.0 (4)
C5—C4—H4	120.1	C13—C12—C10	123.2 (4)
N1—C5—C4	121.0 (5)	C14—C13—C12	119.6 (5)
N1—C5—C6	115.9 (4)	C14—C13—H13	120.2
C4—C5—C6	123.1 (5)	C12—C13—H13	120.2
N2—C6—C7	126.1 (5)	C15—C14—C13	119.4 (5)
N2—C6—C5	114.4 (4)	C15—C14—H14	120.3
C7—C6—C5	119.5 (5)	C13—C14—H14	120.3
C6—C7—H7A	109.5	C14—C15—C16	119.2 (5)
C6—C7—H7B	109.5	C14—C15—H15	120.4
H7A—C7—H7B	109.5	C16—C15—H15	120.4
C6—C7—H7C	109.5	N4—C16—C15	122.0 (5)
H7A—C7—H7C	109.5	N4—C16—H16	119.0
H7B—C7—H7C	109.5	C15—C16—H16	119.0
C6—N2—C8	117.1 (7)	N5—C17—S1	179.2 (5)
